# Primary Hyperparathyroidism in Pregnancy: Successful Parathyroidectomy during First Trimester

**DOI:** 10.1155/2018/5493917

**Published:** 2018-08-06

**Authors:** Niranjan Tachamo, Bidhya Timilsina, Rashmi Dhital, Theresa Lynn, Vasudev Magaji, Ilan Gabriely

**Affiliations:** ^1^Department of Internal Medicine, Reading Hospital, Reading, PA, 19611, USA; ^2^Section of Endocrinology, Reading Hospital, Reading, PA, 19611, USA

## Abstract

Primary hyperparathyroidism in pregnancy can result in significant maternal and fetal complications. When indicated, prompt parathyroidectomy in the early second trimester is considered the treatment of choice. Pregnant patients with primary hyperparathyroidism who have an indication for parathyroidectomy during the first trimester represent a therapeutic challenge. We present the case of a 32-year-old primigravida who presented with symptomatic hypercalcemia from her primary hyperparathyroidism. She remained symptomatic despite aggressive conservative management and underwent parathyroidectomy in her first trimester with excellent outcomes.

## 1. Introduction

Primary hyperparathyroidism (PHP) is rarely diagnosed during pregnancy. It is associated with serious maternal and fetal complications including maternal pancreatitis, nephrolithiasis, hyperemesis, miscarriage, fetal demise, low birth weight, neonatal hypocalcaemia, and tetany. Most cases of PHP during pregnancy are mild, are asymptomatic, and often go undiagnosed [1]. When diagnosed, PHP associated with hypercalcemia should be managed promptly. Surgery (removal of the parathyroid adenoma) in the early second trimester is considered the treatment of choice in selected women [2–4]. We present a case of a primigravida with significant symptomatic hypercalcemia who was diagnosed with PHP and underwent successful parathyroidectomy during her first trimester of pregnancy.

## 2. Case Presentation

A 32-year-old primigravida presented to the Emergency Department (ED) during her 7th week of gestation with complaints of two weeks of progressively worsening intermittent lower abdominal pain. She denied any visual disturbances, headache, nausea, vomiting, constipation or diarrhea, vaginal bleeding, or uterine contractions. Her medical history was significant for a pituitary microadenoma (6.5 × 6 × 5 mm) diagnosed 12 months prior. At that time her serum prolactin was slightly elevated at 35 ng/mL (Ref: 3.34 - 26.72 ng/mL); however, other pituitary hormones were within the normal limits. There was no family history of parathyroid disease, hypercalcemia, nephrolithiasis, or other endocrinopathies except for hypothyroidism affecting her mother. Admission medications included daily prenatal vitamins.

On presentation to the ED, her review of systems was otherwise negative with no genitourinary or gastrointestinal or neurological symptoms. Her vital signs were within normal limits. Her physical examination was unremarkable.

Her blood tests demonstrated hypercalcemia (serum calcium 12.2 mg/dL [Ref: 8.6-10.3 mg/dL], ionized calcium 1.67 mmol/L [Ref: 1.15 - 1.33 mmol/L]), and hyperparathyroidism (PTH 135 pg/mL [Ref: 12-88 pg/mL]). Her serum albumin was 3.2 g/dL (3.5-5.7 g/dL), phosphorus 2.2 mg/dL (Ref: 2.5-5 mg/dL), and magnesium 1.5 mg/dL (Ref: 1.9-2.7 mg/dL). Other relevant labs included a 24-hour urinary calcium of 712 mg/24 hour (Ref: 100-300 mg/24 hr), 25-hydroxyvitamin D 18.5 ng/mL (Deficient if <20 ng/mL), 1,25-dihydroxyvitamin D 94.9 pg/mL (Ref: 19.9-79.3 pg/mL), and thyroid stimulating hormone (TSH) 0.43 uIU/mL (Ref: 0.45-5.33 uIU/mL). Renal ultrasound was unremarkable with no nephrolithiasis or hydronephrosis. Thyroid ultrasound revealed a 28 × 11 × 11 mm hypervascular, heterogeneous mass along the posterior margin of the left thyroid gland. A fine needle aspiration from the mass demonstrated scant cells and was reported as benign cytology. The FNA needle washout resulted in high levels of parathyroid hormone.

**Figure 1:**
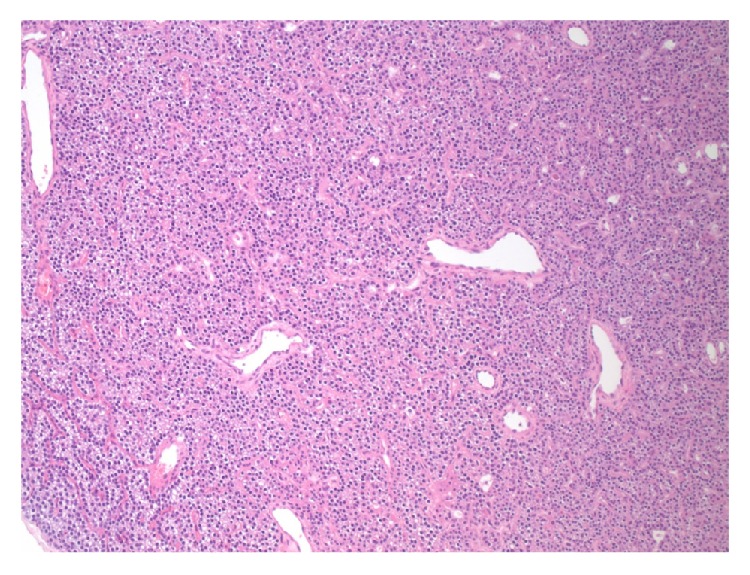
Section of the lesion, showing a diffuse proliferation of cells with loss of the acinar architecture. The lesion show patchy foci of glandular luminal formation (H&E stain, 100x original magnification).

**Figure 2:**
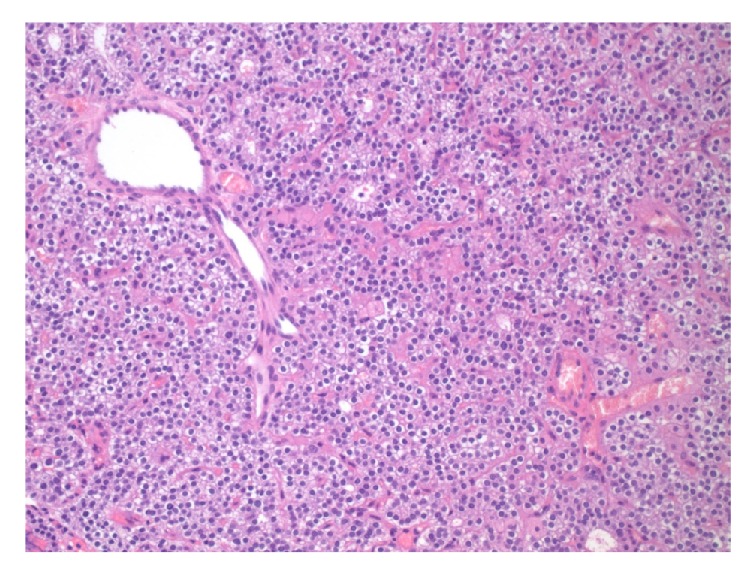
High power view of a representative area, showing that the lesion is composed of uniform-appearing cells, indicating a single cell type proliferation, consistent with adenoma (H&E stain, 400x original magnification).

She was diagnosed with primary hyperparathyroidism and started conservative treatment with IV fluid and magnesium supplements with improvement in her serum calcium levels (11.4 mg/dL). Unfortunately the patient subsequently became symptomatic with nausea, vomiting, and maintaining serum calcium levels of 12 mg/dL despite sufficient hydration. She was started on aggressive hydration (lactated ringers at 125 ml/hr followed by normal saline at 125 ml/hr) continuously until the day of surgery. She received a total of 23 L of intravenous fluids over 10 days; however the serum calcium ranged between 10.6 and 11.6 mg/dL with most values at >11 mg/dL. Just prior to surgery, her serum calcium level was 10.7 mg/dL and her ionized calcium level was 1.38 mmol/L. She underwent left superior parathyroidectomy and the pathology was consistent with a 3.0 × 1.8 × 1.2 cm parathyroid adenoma (Figures 1 and 2). Intraoperative PTH measurement was not performed to reduce the time of anesthesia. Following surgery, her serum calcium and PTH levels normalized. She did not develop hypocalcaemia after surgery. In subsequent follow-up weekly visits after discharge, her serum calcium and PTH levels have been within the normal limits.

With her history of pituitary adenoma and a large parathyroid adenoma, multiple endocrine neoplasia type 1 (MEN1) was considered. However, direct DNA testing for MEN1, RET, AIP, and CDKN1B gene mutations were negative.

## 3. Discussion

Primary hyperparathyroidism is a common endocrine disorder and has a prevalence estimate of about one to seven cases per 1000 adults. The true incidence of PHP is difficult to estimate; however it is considered to vary between 0.4 and 21.6 cases per 100000 person-years [5]. The incidence increases with age, and it is two to three times more common in women [5], with peak incidence in women aged 50-60. The exact incidence of PHP in pregnancy is unknown, and most data derives from case reports.

The exact cause of PHP is not known. It was demonstrated that irradiation to the neck (for benign conditions or in survivors of an atomic bomb) was associated with a significantly increasing incidence of PHP [6]. MEN 1 and MEN 2 are examples of genetic mutations associated with PHP [4].

PHP is rarely diagnosed in pregnancy as many cases are mild and asymptomatic. Moreover, many of the nonspecific symptoms of pregnancy, such as nausea, vomiting, fatigue, and constipation, are similar to those associated with hyperparathyroidism, which leads to missed or delayed diagnosis [2]. It is considered that as many as 80% of cases of PHP during pregnancy may be asymptomatic. However, untreated PHP can be associated with significant and serious maternal (e.g., hypercalcemic crisis, pancreatitis, nephrolithiasis, and preeclampsia) and fetal (e.g., neonatal tetany, hypoparathyroidism, stillbirth, and miscarriage) complications which may be as high as 67% and 80% respectively [1, 4]. This may be an overestimate, taking into account that most cases are mild, asymptomatic and undiagnosed, and only clinically evident cases are likely to be reported [4].

Moreover, a large retrospective study by Abood et al. did not find any significant difference in pregnancy outcomes between patients with PHP during pregnancy and age-matched controls [7]. Norman et al., however, showed that hyperparathyroidism in pregnancy had a 3.5 times higher rate of abortion when compared to the general population. As expected, the rate of complications increased with the escalating levels of maternal serum calcium [3].

The diagnosis of PHP during pregnancy is similar to nonpregnant patients with the exception that physiological changes in pregnancy (plasma volume expansion) result in low total serum calcium level; the upper limit of normal total calcium in pregnancy is considered 9.5 mg/dL [1]. Alternatively, ionized calcium does not change significantly with pregnancy and may represent a better assay to assess serum calcium levels during pregnancy [2, 4].

Neck ultrasound is used to identify the parathyroid adenoma (sensitivity 69%, specificity 94%) as nuclear scans are contraindicated in pregnancy due to concern for fetal risk from ionizing radiation [2]. Fine needle aspiration and needle washout for PTH are not routinely recommended (it carries a particular risk in patients with parathyroid cancer). However, since surgery in the first trimester carries a substantial risk, we wanted to ensure that the nodule identified in the thyroid ultrasound was indeed a parathyroid adenoma prior to subjecting the patient to surgery. In the overall risk calculation, we took into account the facts that the prevalence of parathyroid carcinoma in the general population is very low and it usually occurs in the higher age group. Furthermore, parathyroid carcinoma is associated with significantly higher serum calcium and PTH levels. Thus, taking into account the patient's demographic data, the clinical presentation and serum calcium, and PTH level, we considered that her risk of having parathyroid carcinoma was very low.

In the differential diagnosis, familial hypocalciuric hypercalcemia and hereditary syndromes such as MEN-1 and MEN-2 should always be considered [2], both of which were ruled out in our patient.

There are no clear guidelines for management of PHP during pregnancy. Treatment should be tailored based on symptoms, severity of hypercalcemia, gestational age, and the risk-benefit assessment [8].

Mild cases of asymptomatic PHP in pregnancy may be managed conservatively with a low calcium diet, adequate hydration, and close monitoring of serum calcium. A small dose of vitamin D may be supplemented cautiously in deficient patients to decrease PTH levels, as well as lower the risk of hungry bone syndrome postoperatively if parathyroidectomy is planned. However, excess and rapid supplementation can lead to aggravation of the hypercalcemia and hypercalcemic crisis [4]. In more severe cases, cinacalcet and calcitonin may be used with temporary results. Bisphosphonates are not indicated in pregnancy because of the risk of fetal bone toxicity and modeling abnormality [2].

Parathyroidectomy remains the only curative treatment. In multiple reports, conservative management was shown to have higher fetal complications when compared to parathyroidectomy in pregnancy [9, 10]. The current recommendation is to perform parathyroidectomy during the second trimester of pregnancy due to incomplete organogenesis in first trimester, and risk of preterm labor in the third trimester [4]. While there are multiple reports of successful second and third trimester parathyroidectomy, there are only limited data on parathyroidectomy during the first trimester. In a review by Carella et al. [11], parathyroidectomies were reported for all trimesters with 34/38 live births (7/7 in first trimester, 17/18 in second trimester, 9/12 in third trimester, and 1/1 unspecified). When feasible, a minimally invasive approach rather than open procedures and intraoperative PTH monitoring is recommended [2]. Our patient underwent minimally invasive parathyroidectomy; however, intraoperative PTH monitoring was not performed in order to reduce time under anesthesia.

In a retrospective study, Norman et al. [3] reported that 71% of the study participants had prior history of miscarriage and were able to achieve 100% successful pregnancy outcome after parathyroidectomy with no subsequent maternal or fetal complications, demonstrating the efficacy of parathyroidectomy in PHP during pregnancy. Furthermore, the study emphasized the detrimental effects of increasing maternal serum calcium levels on pregnancy outcome, with 73% pregnancy loss when maternal serum calcium levels were 11.4 mg/dL or higher. While pregnancy loss occurred even with mild elevations in serum calcium at 10.7 mg/dL, fetal death was more likely to occur when the serum calcium level was 11.4 mg/dL or higher. The study also showed that the majority of pregnancy loss occurred in the late first or early second trimester with the majority of pregnancy losses at 10-15 weeks gestation. Hence, the authors proposed surgical intervention in the late first or early second trimester rather than waiting until mid-second trimester, especially with elevated maternal serum calcium levels of >11.4 mg/dL and prior history of miscarriage [3]. Our patient underwent parathyroidectomy in the first trimester, having both symptomatic hypercalcemia and a serum calcium level >11.4 mg/dL (uncontrollable with conservative treatment).
